# Primary decompressive craniectomy in patients with large intracerebral hematomas due to aneurysmal subarachnoid hemorrhage

**DOI:** 10.1007/s00701-024-06221-9

**Published:** 2024-08-10

**Authors:** Fabian Wenz, Andreas Ziebart, Katharina A. M. Hackenberg, Gabriel J. E. Rinkel, Nima Etminan, Amr Abdulazim

**Affiliations:** Department of Neurosurgery, University Hospital Mannheim, Medical Faculty Mannheim, University of Heidelberg, Theodor-Kutzer-Ufer 1-3, 68167 Mannheim, Germany

**Keywords:** Complications, Decompressive craniectomy, Delayed cerebral ischemia, Intracerebral hemorrhage, Subarachnoid hemorrhage

## Abstract

**Background:**

Decompressive craniectomy (DC) can alleviate increased intracranial pressure in aneurysmal subarachnoid hemorrhage patients with concomitant space-occupying intracerebral hemorrhage, but also carries a high risk for complications. We studied outcomes and complications of DC at time of ruptured aneurysm repair.

**Methods:**

Of 47 patients treated between 2010 and 2020, 30 underwent DC during aneurysm repair and hematoma evacuation and 17 did not. We calculated odds ratios (OR) for delayed cerebral ischemia (DCI), angiographic vasospasm, DCI-related infarction, and unfavorable functional outcome (extended Glasgow Outcome Scale 1–5) at three months. Complication rates after DC and cranioplasty in the aneurysmal subarachnoid hemorrhage patients were compared to those of all 107 patients undergoing DC for malignant cerebral infarction during the same period.

**Results:**

In DC versus no DC patients, proportions were for clinical DCI 37% versus 53% (OR = 0.5;95%CI:0.2–1.8), angiographic vasospasm 37% versus 47% (OR = 0.7;95%CI:0.2–2.2), DCI-related infarctions 17% versus 47% (OR = 0.2;95%CI:0.1–0.7) and unfavorable outcome 80% versus 88% (OR = 0.5;95%CI:0.1–3.0). ORs were similar after adjustment for baseline predictors for outcome. Complications related to DC and cranioplasty occurred in 18 (51%) of subarachnoid hemorrhage patients and 41 (38%) of cerebral infarction patients (OR = 1.7;95%CI:0.8–3.7).

**Conclusions:**

In patients with aneurysmal subarachnoid hemorrhage and concomitant space-occupying intracerebral hemorrhage, early DC was not associated with improved functional outcomes, but with a reduced rate of DCI-related infarctions. This potential benefit has to be weighed against high complication rates of DC in subarachnoid hemorrhage patients.

## Introduction

Aneurysmal subarachnoid hemorrhage (ASAH) is a life-threatening subtype of stroke with a case-fatality rate of 44% in Europe [15]. Concomitant space-occupying intracerebral hematoma is present in 21% of all cases and is associated with a worse prognosis [7, 21]. In a clinical trial published in 1988, immediate hematoma evacuation and microsurgical aneurysm repair reduced case fatality, compared to postponed surgical treatment [8]. However, the functional outcome in such patients remains poor, which probably relates to high intracranial pressure in these patients even after the removal of the hematoma [7, 21].

Decompressive craniectomy (DC) is an effective treatment to relieve increased intracranial pressure and improve local perfusion in the setting of malignant cerebral infarction [2, 3]. For patients with ASAH, DC is considered in case of space-occupying hematomas or extensive brain swelling [10, 14, 19]. Apart from its potential beneficial effects, DC also carries a risk for complications from the procedure itself and from the inherent cranioplasty performed 6–12 weeks after decompression [12, 13]. The risk of complications from DC may be different in patients with ASAH and concomitant hematoma from that in patients with malignant cerebral infarction. On the one hand, patients with malignant cerebral infarction are generally older than patients with ASAH, which may imply a lower risk of complications in ASAH patients. On the other hand, patients with ASAH often have more intracranial and systemic complications, which may imply a higher risk of complications. The literature on DC in patients with ASAH is scarce and has conflicting results. Studies available to date have limitations such as small sample sizes and lack of control groups [4, 5, 16, 23]. Thus, in contrast to the evidence for patients with malignant cerebral infarction, the risk–benefit ratio of DC in ASAH patients remains uncertain.

We aimed to determine the effect of early DC at time of surgical aneurysm repair and hematoma evacuation. We therefore compared clinical outcomes in patients with DC to similar patients without DC and assessed procedure-related complications in patients undergoing DC after ASAH and space occupying intracerebral hematoma. These complications were compared to complications occurring in patients with DC for malignant cerebral infarction.

## Materials and methods

### Patients

Out of 601 consecutive patients with ASAH who were admitted to our hospital between January 2010 to December 2020, we included all 47 patients with space-occupying intracerebral hematoma, which was defined as blood clot volume > 20 ml, who underwent surgical clipping and hematoma evacuation. Of these patients, 30 patients were treated with and 17 without DC at time of surgical aneurysm treatment and hematoma evacuation. For comparison of the surgical complication rates related to DC and cranioplasty, we additionally retrieved data from all 107 patients who underwent DC for malignant cerebral infarction within the same period.

### Data collection

We extracted baseline data from the clinical files, including age, sex, clinical condition of admission according to the WFNS (World Federation of Neurosurgical Societies) classification [17] and aneurysm characteristics (site and size). Midline shift and blood clot volume were measured with the Elements software Smart Brush® (version 3.0.0.92, Brainlab).

Clinical DCI was defined as a decrease in level of consciousness, a new focal neurological deficit, or both with other causes excluded [20].

Angiographic vasospasm was defined as narrowing of the arterial diameter of at least 50% from baseline on digital subtraction angiography or CT angiography.

DCI-related cerebral infarctions were considered in the presence of new hypodensities within 6 weeks after the primary ASAH ictus but not within 48 h after aneurysm repair and therefore not attributable to the surgical treatment and/or the intraparenchymal hematoma [20].

Functional outcome was assessed using dichotomized extended Glasgow Outcome Scale (eGOS) at 3 months and 1 year after the primary ASAH ictus (unfavorable: eGOS 1–5 and favorable: eGOS 6 – 8).

We also retrieved the rate of secondary DCs in the 17 DC- patients.

Postoperative complications were considered for analysis when a surgical revision was necessary. Postoperative complications were dichotomized into complications associated to early DC versus complications from subsequent cranioplasty.

### Clinical management

In all patients with ASAH and intracerebral hematoma who underwent immediate surgical aneurysm repair with clipping and hematoma evacuation, we used a frontotemporal approach. The decision to perform DC at time of aneurysm treatment was made on an individual basis considering radiological factors such as initial extent of midline shift and intraoperative findings, e.g. extensive brain swelling. When DC was chosen by the treating neurosurgeon, it was performed as an extended frontotemporal craniectomy with subsequent star-shaped dural opening. Postoperatively, patients were further monitored on the intensive care unit for at least 14 days after ictus and underwent regular neurological evaluation. Blood pressure was continuously monitored and maintained above a systolic blood pressure of 130 mmHg in all patients. In case of an external ventricular drain placement, intracranial pressure was continuously monitored, and cerebrospinal fluid was discontinuously drained at an upper threshold of 18 mmHg. Between 2010 and August 2015, management of delayed cerebral ischemia (DCI) was based primarily on the detection of vasospasm and subsequent continuous administration of nimodipine within the internal carotid artery, while a risk-based, escalating approach was established as of September 2015 [1].

Cranioplasty was as per standard performed after 3 months after DC.

For patients with malignant cerebral infarction, decompressive hemicraniectomy was performed by removing a bone flap of at least 12 cm in diameter, including portions of the frontal, parietal, temporal, and occipital skull, followed by a star-shaped dural opening. After surgery, patients were transferred to the intensive care or stroke unit, dependent on their clinical condition.

### Statistical analysis

Statistical analyses were performed using R version 3.6.2 [The R Foundation]. Continuous data were presented as mean with standard deviation (SD). Categorical data are presented as numbers (%). To analyze the effect of DC during surgical aneurysm repair and intracerebral hematoma evacuation on functional outcome and secondary brain injury a logistic regression model was used. We calculated crude odds ratios (OR) with confidence intervals (CI) and adjusted for age, WFNS grade, midline-shift and clot volume, and the rate of external ventricular drain application. For the analysis of the procedure related complications, we calculated only crude odds ratios.

## Results

The patient demographics are highlighted in Table 1. In terms of sex distribution, the groups with and without DC were comparable, but patients with DC were slightly older and more often in a poor clinical condition according to WFNS grade and received an external ventricular drain during primary treatment and ventriculoperitoneal shunt more often. The clot volumes of the intracerebral hematoma and the midline shift were comparable in both groups. In the DC- cohort, 5 patients received a secondary DC due to refractory elevated intracranial pressure. Cranioplasty was performed in 26 patients within a median of 104 days after ictus (IQR 70–149). Of all patients with malignant cerebral infarction, 49% were female and the mean age was 58 years.

**Table 1:** Patient characteristics

Characteristics	DC +	DC-
Number of patients	30	17
Female (%)	26 (87)	15 (88)
Mean age (years ± SD)	59 ± 9	57 ± 10
WFNS grade (%)		
I	0 (0)	0 (0)
II	1 (3)	0 (0)
III	6 (20)	6 (35)
IV	9 (30)	6 (35)
V	14 (47)	5 (29)
Aneurysm site (%)		
MCA	29 (97)	12 (71)
ACA	1 (3)	0 (0)
PCOM	0 (0)	5 (29)
Aneurysm side (%)		
Left	10 (33)	7 (41)
Right	19 (63)	10 (59)
Midline	1 (3)	0 (0)
Radiological measurements		
Clot volume (ml ± SD)	50 ± 19	46 ± 18
Midline-shift (mm ± SD)	7 ± 4	6 ± 2
CSF diversion (%)		
EVD placement	27 (90)	7 (41)
VP-shunt placement	16 (53)	3 (18)

Baseline characteristics of patients treated with (DC +) and without (DC-) decompressive craniectomy; *WFNS* World Federation of Neurosurgical Societies; *MCA* Middle cerebral artery; *ACA* Anterior cerebral artery; *PCOM* Posterior communicating artery; *SD* standard deviation; *EVD* External ventricular drain; *VP-shunt* ventriculoperitoneal shunt

### Outcomes after decompressive craniectomy and cranioplasty

**Fig. 1:**
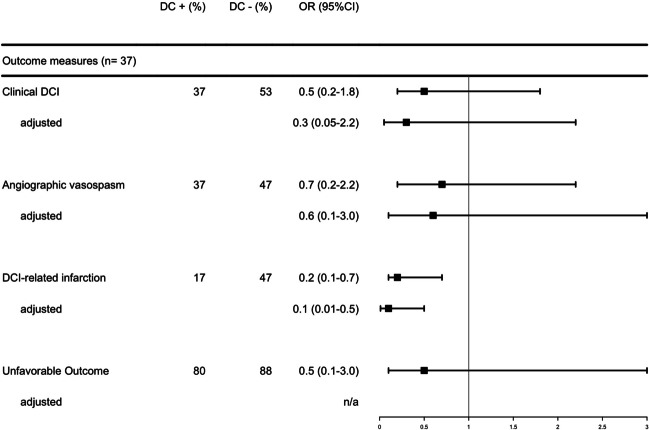
Effect of decompressive craniectomy on radiological and functional outcome. Radiological and functional outcome of patients treated with (DC +) and without (DC-) decompressive craniectomy; Unfavorable outcome is extended Glasgow outcome scale of 1 – 5 3 months after discharge; Multivariate analysis was adjusted for age, WFNS grade, midline-shift, clot volume and EVD placement; OR = odds ratio; CI = confidence interval; DCI = delayed cerebral ischemia

**Fig. 2:**
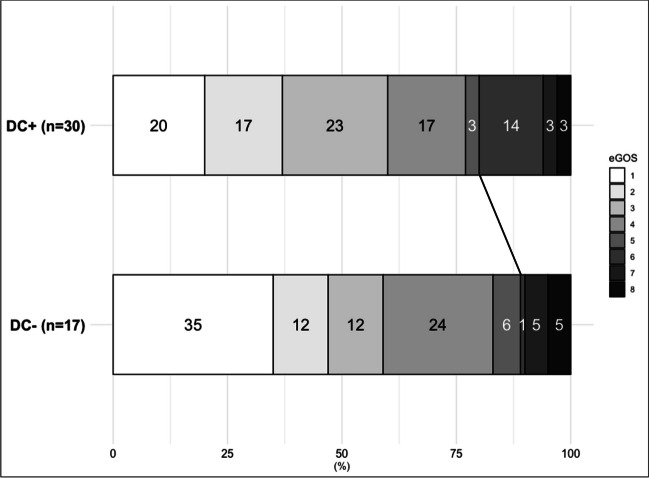
Stacked histograms of functional outcome. Functional outcome (extended Glasgow Outcome Scale) after 3 months of patients treated with (DC +) and without (DC-) decompressive craniectomy

Clinical DCI occurred in 11 (37%) patients with and in 9 (53%) patients without DC (OR = 0.5, 95% CI = 0.2 – 1.8). Angiographic vasospasm was present in 11 (37%) patients with and 8 (47%) patients without DC (OR = 0.7, 95% CI = 0.2 – 2.2), and DCI-related infarctions were evident at discharge in 5 (17%) patients with and in 8 (47%) patients without DC (OR = 0.2, 95% CI = 0.1 – 0.7). Unfavorable outcome at 3 months occurred in 24 (80%) patients with and 15 (88%) patients without DC (OR = 0.5, 95% CI = 0.1 – 3.0). Results were essentially the same after adjustment (Table 2, Figs. 1 and 2). One-year outcome was available for 21 (70%) DC + patients and 12 (71%) DC- patients. In the DC + group, two patients improved during this period (eGOS 3 to 4 and 6 to 7) and one patient died in an external hospital due to pneumogenic sepsis, but there were no changes in the dichotomized outcome results. For those patients who underwent cranioplasty, outcomes were available for 25 patients (96%) and there were no changes compared to outcomes at 3 months.

**Table 2:** Outcome measurements

Outcomes	DC + n (%)	DC- n (%)	Unadjusted OR (95% CI)	Adjusted OR (95% CI)
Clinical DCI	11 (37)	9 (53)	0.5 (0.2 – 1.8)	0.3 (0.05 – 2.2)
Vasospasm	11 (37)	8 (47)	0.7 (0.2 – 2.2)	0.6 (0.1 – 3.0)
DCI-related infarction	5 (17)	8 (47)	0.2 (0.1 – 0.7)	0.1 (0.01 – 0.5)
Unfavorable outcome	24 (80)	15 (88)	0.5 (0.1 – 3.0)	n.a

Outcome measurements of patients treated with (DC +) and without (DC-) decompressive craniectomy; *OR* odds ratio; *CI* confidence interval; *DCI* delayed cerebral ischemia; Unfavorable outcome is extended Glasgow outcome scale of 1 – 5 3 months after discharge; Multivariate analysis was adjusted for age, WFNS grade, midline-shift, clot volume and EVD placement; for unfavorable outcome no adjustment (n.a.) was done because of the wide confidence interval for the unadjusted OR

### Complications after decompressive craniectomy and cranioplasty

Complications due to DC and cranioplasty occurred in 51% (18) of the subarachnoid hemorrhage patients, and in 38% (41) of the cerebral infarction patients (OR = 1.7, 95% CI = 0.8 – 3.7). The complication rate related specifically to DC was 43% (15 patients) in the subarachnoid hemorrhage group and 29% (31 patients) in the cerebral infarction group (OR = 1.8, 95% CI = 0.8 – 4.1). In both groups the most frequent complication was wound healing disorder (subarachnoid hemorrhage: 6 patients (17%), cerebral infarction: 17 patients (16%)). The complication rate after cranioplasty was 31% (8 patients) in the subarachnoid hemorrhage group and 22% (16 patients) in the cerebral infarction group (OR = 1.6, 95% CI = 0.6 – 4.4). The different types of complications were similar in both groups (Table 3).

**Table 3:** Postoperative complications

Procedure and complications	ASAH	Cerebral Infarction	OR (95% CI)
DC performed	35	107	
Complications related to DC (%)	15 (43)	31 (29)	1.8 (0.8 – 4.1)
Wound healing problems	6 (17)	17 (16)	
Infection	2 (6)	6 (6)	
Postoperative hemorrhage	5 (14)	8 (8)	
CSF fistula	3 (9)	7 (7)	
Cranioplasty performed (%)	26 (74)	74 (69)	
Complications related to cranioplasty (%)	8 (31)	16 (22)	1.6 (0.6 – 4.4)
Wound healing problems	1 (4)	5 (7)	
Infection	1 (4)	4 (5)	
Postoperative hemorrhage	3 (12)	3 (4)	
Dislocation implant	1 (4)	2 (3)	
Bone flap osteolysis	3 (12)	3 (4)	

Postoperative complications related to decompressive craniectomy (DC) and cranioplasty in patients with aneurysmal subarachnoid hemorrhage (ASAH) and cerebral infarction; *OR* odds ratio; *CI* confidence interval; *CSF* cerebrospinal fluid

## Discussion

Patients who underwent early DC during surgical aneurysm repair and intracerebral hematoma evacuation had lower rates of DCI-associated infarctions than patients without early DC, but this did not translate into improved functional outcome. Apart from this potential beneficial effect, DC carried a substantial risk for surgical complications, which was distinctly higher than in patients undergoing the same procedures after malignant cerebral infarction.

The proportion of patients with a good outcome is somewhat lower than in previous series where proportions of favorable outcome after primary DC are reported in around 35% of patients [5, 16]. The differences between our and the other studies can be explained by different outcome scales and dichotomies of these scales used in the different studies. Despite these differences, the results from our studies in combination with previous studies show that patients in a very poor clinical condition from a ruptured intracranial aneurysm with a large, space occupying lesion can have a good outcome after immediate DC. We found a reduced risk of DCI-related infarction in patients undergoing immediate DC. In a study wherein patients with middle cerebral artery aneurysms and concomitant intracerebral hematoma and immediate DC were compared to no DC, no difference in cerebral infarction was found between the two groups. In that study, however, all causes for cerebral infarction were pooled, whereas we focused specifically on DCI-related infarction [23].

DC is an invasive procedure with a high complication rate, but a reasonable treatment option for patients with a decreasing level of consciousness from brain swelling after malignant cerebral infarction [9, 11, 18]. A large meta-analysis reported a rate of postoperative hemorrhagic complications of 12% for DC and 3.6% for cranioplasty, including all underlying indications for initial DC [12]. Our results show hemorrhagic complications of 14% (subarachnoid hemorrhage) and 8% (cerebral infarction) for DC and 12% (subarachnoid hemorrhage) and 4% (cerebral infarction) for cranioplasty, which compares well with the literature. The same applies to the reported rates of CSF fistula after DC (6%), infectious complications (7% after DC and 6% after cranioplasty) and bone flap osteolysis after cranioplasty (10%) [12]. However, our results show a high overall complication rate, with wound healing problems being a major contributory factor. This may be because the indication for revision surgery for superficial wound complications is highly heterogeneous between institutions. In addition, complication rates and definitions of postoperative complications vary widely between reported series. With some including only individual complications such as rebleeding [23] and others including non-surgical complications such as seizures or hydrocephalus, a direct comparison of overall complication rates appears to be limited [22].

Our findings can be interpreted in different ways. DC in combination with hematoma evacuation may result in a better microcirculation and local perfusion than hematoma evacuation alone. Previous studies have shown improvement of perihematomal hemodynamics after surgical removal of spontaneous lobar intracerebral hematomas [6]. DC reduces intracranial pressure, which can affect the cerebral perfusion pressure and thus local perfusion [2]. Additionally, the higher rate of acute CSF diversion in the DC + cohort through perioperative application of an external ventricular drain may have further reduced the intracranial pressure and improved local perfusion. Since DCI may in part be explained by microcirculatory disturbances, the combined effect of DC and hematoma evacuation may explain the reduced rate of clinical DCI and DCI-related cerebral infarction in patients with DC.

The lack of improved clinical outcome after DC despite a reduction in DCI related infarction may relate to the overall small proportion of patients having a good outcome, which limits the statistical power of our cohort. Thus, even though in the end DC may improve outcome, we could not find it in our study, which is a difference to the indication for malignant cerebral infarction, where DC is an effective treatment. One explanation for this difference regarding effects according to the indication of DC may be the higher complication rate in patients with ASAH than patients with malignant cerebral infarction. This higher complication rate may be explained by the more extensive surgical procedures used to treat the aneurysm, such as brain retraction or opening of the cerebrospinal fluid spaces by opening the cisterns or ventricles. In addition, patients after ASAH are in a more severe clinical condition with a much longer stay on the intensive care unit and potential involvement of different organ systems such as acute cardiorespiratory, gastrointestinal or infectious pathologies than patients with malignant cerebral infarction.

Our study has strengths. First, to the best of our knowledge this is the first case–control study investigating the effect of DC during aneurysm treatment on DCI-specific complications in patients with ASAH and space-occupying intracerebral hemorrhage. Second, our study is much larger than earlier studies on ASAH patients. In addition, we compared the risk of DC related complications in SAH patients with that in patients with malignant cerebral infarction, which showed different risks.

However, we acknowledge several limitations. Although the number of patients is larger than in previous studies, it may still be too small to detect a meaningful effect on clinical outcome. Moreover, the retrospective, non-randomized and unblinded study design is always subject to bias by indication for DC, even though the baseline characteristics were reasonably comparable in both groups. The imbalance in the rate of acute EVD treatment between the DC + and DC- cohort may imply that patients in the DC- cohort were undertreated with respect to acute hydrocephalus. In fact, this would have had an impact on outcome rates. However, we have calculated the relative bicaudate index (RBCI) as an objective parameter for enlarged ventricles and hydrocephalus. The median RBCI in patients that did not receive an external ventricular drain was 0.53 (IQR 0.46 – 0.75) indicating no hydrocephalus. This is also reflected in the lower rate of permanent shunt treatment in the DC- cohort (DC + : 53% vs. DC-: 18%). Although, acute CSF diversion, without acute hydrocephalus being present, may be beneficial to improve local perfusion, we do not assume that the imbalance in the rate of EVD treatment introduced significant bias by indication. In addition, the influence of comorbidities between the DC + and DC- cohort was not assessed. However, the presence of comorbidities will mainly impact the decision for or against aneurysm repair and not for or against decompressive craniectomy. Since all patients in our cohort were considered fit enough to undergo aneurysm repair, we do not expect a significant impact on outcome between the DC + and DC-cohorts because of a difference in comorbidities. Finally, we did not perform preoperative MRI or CT perfusion imaging as this is logistically not feasible in this patient subset. Therefore, we cannot make conclusions about the tissue at risk, especially in the proximity of the hematoma, that might be salvaged by hemicraniectomy.

## Conclusions

The reduced risk of DCI related infarctions in patients with ASAH and space-occupying intracerebral hemorrhage treated with primary DC suggests a beneficial effect of this procedure. We will therefore continue to consider this treatment in such patients, but the potential benefits have to be weighed against the high risk of complications of the procedure. Decision have therefore to be made on an individual basis. Further studies should address how to reduce the risk of complications of immediate DC in these patients and should preferably have an allocation between treatment and control group that is less prone to bias than the decision of the treating physician or surgeon.

## Data Availability

The dataset analyzed in this study is available from the corresponding author on reasonable request.

## Abbreviations

ASAH: Aneurysmal subarachnoid hemorrhage
CI: Confidence interval
DC: Decompressive craniectomy
DCI: Delayed cerebral ischemia
eGOS: Extended Glasgow Outcome Scale
OR: Odds ratio
SD: Standard deviation
WFNS: World Federation of Neurosurgical Societies
